# UHPLC-Triple-TOF-MS Characterization, Antioxidant, Antimicrobial and Antiproliferative Activity of Raspberry (*Rubus idaeus* L.) Seed Extracts

**DOI:** 10.3390/foods12010161

**Published:** 2022-12-28

**Authors:** Boško Marić, Biljana Abramović, Nebojša Ilić, Marija Bodroža-Solarov, Branimir Pavlić, Michał Oczkowski, Jacek Wilczak, Dragana Četojević-Simin, Ljubiša Šarić, Nemanja Teslić

**Affiliations:** 1 University of Novi Sad, Institute of Food Technology, Bulevar cara Lazara 1, 21000 Novi Sad, Serbia; 2 University of Novi Sad, Faculty of Science, Department of Chemistry, Biochemistry and Environmental Protection, Trg Dositeja Obradovića 3, 21000 Novi Sad, Serbia; 3 University of Novi Sad, Faculty of Technology, Bulevar cara Lazara 1, 21000 Novi Sad, Serbia; 4 Warsaw University of Life Sciences (WULS-SGGW), Institute of Human Nutrition Sciences, Department of Dietetics, Nowoursynowska 159c, 02-776 Warsaw, Poland; 5 Warsaw University of Life Sciences (WULS-SGGW), Institute of Veterinary Medicine, Department of Physiological Sciences, Nowoursynowska 159, 02-776 Warsaw, Poland; 6 Singidunum University, Department of Pharmacy, Danijelova 32, 11000 Belgrade, Serbia

**Keywords:** bioactivity, raspberry seeds, polyphenols profile, antioxidant activity, antiproliferative activity

## Abstract

The primary aim of this experiment was to investigate the bioactivity potential and polyphenolic profile of defatted raspberry seeds (DRS) extracts from three varieties (Willamette, Meeker, and Polka) using the in vitro tests HPLC-DAD and UHPLC-Triple-TOF-MS. Extracts were obtained using ultrasound-assisted extraction (UAE) or hydrolysis. The antioxidant activity of the extracts was tested using 1,1-diphenyl-2-picrylhydrazyl (DPPH), 2,2′-azinobis-(3-ethylbenzothiazoline-6-sulfonic) cation (ABTS), and ferric reducing antioxidant power (FRAP) assays. Furthermore, the extracts were tested for antimicrobial activity using the disk diffusion method for four bacterial cultures (*Staphylococcus aureus*, *Escherichia coli*, *Listeria monocytogenes*, and *Salmonella enterica* subsp. *enterica* Enteritidis). In vitro antiproliferative activity was tested using cervical carcinoma (HeLa), breast adenocarcinoma (MCF7), and fetal lung (MRC-5) human cell lines. In total, 32 phenolic compounds were detected in DRS extracts. A small quantity of ellagic acid (EA) was in free form, while EA content increased after the hydrolysis process. The extracts from the Meeker variety exhibited the highest antioxidant activity, analyzed with DPPH and FRAP assays, while extracts from the Polka variety had the highest activity towards ABTS•^+^ radical scavenging activity. The UAE samples expressed higher antiproliferative activity in comparison to hydrolysis extracts. The results indicate that DRS extracts have certain bioactivity, and their use in the food, cosmetic, and pharmaceutical industries is recommended.

## 1. Introduction

The exponential growth of the world population could lead to nutrition insecurity in less developed countries, necessitating an increase food demand. Malnutrition further leads to an increased risk of negative health effects, which may harm human health. One of the solutions to this problem is to create sustainable food systems, which include the production of functional foods, and thereby address the problem of undernutrition. One way to create functional food is to valorize the by-products from the agro-food sector that are considered waste [1] but are rich in bioactive compounds. Due to the suitable climate, Serbia is one of the world’s largest producers and exporters of raspberries (*Rubus idaeus* L.), which are partly processed by the fruit industry. Thus, large amounts of seeds are discarded annually during the processing of fresh raspberries into juices, jams, and jellies, and these seeds are under-exploited or wasted as by-products.

Previous research has shown that raspberry seeds could be valorized as a significant source of bioactive compounds [2,3]. Raspberry seeds are a significant source of high-quality oil, especially high concentrations of ω-3 polyunsaturated fatty acids (PUFA) and good ω-6/ω-3 ratio, which may provide potential health benefits in preventing heart disease, cancer, hypertension, and autoimmune disorders [4,5,6]. This oil may be recovered from seeds using supercritical CO_2_ extraction [7], cold pressing, or other extraction techniques [4].

Furthermore, after oil extraction, residue can be valorized as a rich source of polar, polyphenolic compounds, such as ellagic acid (EA) and its conjugates, ellagitannins, and ellagic acid glycosides [8]. Previous research has reported that a large amount of EA in raspberry is contained in seeds in the form of ellagitannins [9]. The properties of EA and other phenolic acids that are beneficial to health are well known. Polyphenols are known to exhibit antioxidant, antimicrobial, antiproliferative, and anti-inflammatory properties [10,11]. Moreover, EA can be used in obesity prevention [12]. Polyphenolic extracts have been proven to inhibit the development of obesity and hyperlipidemia by inhibiting pancreatic lipase activity and suppressing energy intake [13]. Previous research has demonstrated that intake of food that is rich in EA can prevent chronic diseases, such as cardiovascular and neurodegenerative diseases [14,15].

High intakes of polyphenol-rich fruits, vegetables, and whole grains are directly associated with a reduced risk of many non-communicable chronic diseases [16], which are associated with increased oxidative stress and disturbed redox balance in the body. Phytochemicals, especially polyphenols, primarily contribute to the overall antioxidant activity in plants [17], including fruits. Polyphenols also exert their antimicrobial effect by inhibiting the growth of microorganisms, separating metal ions that are critical for microbial growth and metabolism, or inhibiting critical bacterial membrane functions, such as ion channels and proteolytic activity [18].

Previous studies have shown that EA exhibits anticarcinogenic properties, such as induction of cell cycle arrest and apoptosis, as well as inhibition of tumor formation and tumor growth in animals [19]. Inhibition of the formation of various cancers by EA occurs through several mechanisms.

The main objectives and novelty of this research were: (I) development of a “green” extraction process (i.e., ultrasound-assisted extraction in combination with aqueous ethanol) for valorization of polyphenol-rich raspberry seeds that are currently discarded as waste in Serbia; (II) characterization of extracts in terms of polyphenolic profile using UHPLC-Triple-TOF-MS; (III) determination of antimicrobial, antioxidant, and antiproliferative activity of the obtained extracts; (IV) examination of three raspberry varieties commonly cultivated in Serbia with the aim of elucidating diversity in polyphenolic profile and in vitro activities between varieties.

## 2. Materials and Methods

### 2.1. Material

The raspberry seeds of three cultivars (Willamette, Polka, and Meeker) were kindly donated by Mondi Lamex d.o.o., a company from Kraljevo, Serbia, in 2019. The samples were milled on a laboratory mill (Glen Mills, Clifton, NJ, USA) to achieve a granulation of 200–400 µm. Moisture content (5.71 ± 0.19%) was determined using gravimetrical AOAC method 950.46/2006, also known as the ‘oven-dry’ method [20].

The raspberry seeds of all three cultivars were defatted using a laboratory extractor (NOVA-Swiss, High-pressure extraction plant 565.0156; Nova Werke Ltd., Effertikon, Swiss). To optimize the extraction parameters and extract the maximum quantity of oil, 15 experiments were performed according to response surface methodology (RSM). The examined parameters were pressure (250–350 bar), temperature (40–60 °C), and CO_2_ flow (0.2–0.4 kg/h). The outcomes were optimal parameters for oil extraction: pressure 340 bar, temperature 51 °C, and 0.4 kg/h CO_2_ flow [21].

### 2.2. Chemicals and Reagents

Ethanol (98%) (Sigma-Aldrich, Steinhaus, Germany), methanol (99.9%), and hydrochloric acid (37%) (Fisher Scientific, Loughborough, Great Britain) were used for polyphenol extraction. Formic acid (HPLC gradient), analytical standard of EA (≥95.0%) (Sigma-Aldrich, Steinheim, Germany), and acetonitrile (HPLC gradient) (Carlo Erba, Chaussée du Vexin, France) were used to determine EA content. The following chemicals were used to determine antioxidant activity: 1,1-diphenyl-β-picrylhydrazyl (DPPH), 2,2′-azino-bis (3-ethylbenzothiazoline-6-sulfonic acid) diammonium salt (ABTS), 2,4,6-tripyridyl-s-triazine (TPTZ), iron(III) chloride (p.a.), 6-hydroxy-2,5,7,8-tetramethylchroman-2-carboxylic acid (trolox) (98%) (Sigma-Aldrich, Steinheim, Germany), potassium persulfate (99%) (Lach-Ner, Neratovice, Czech Republic), and hydrochloric acid (37%) (Fisher Scientific, Loughborough, UK). Acetate buffer was made using sodium acetate (99%) and glacial acetic acid (98%) (Sigma-Aldrich, Steinheim, Germany). Nutrient agar (Himedia, Maharashtra, India) and Mueller–Hinton agar (LabM, Lancashire, UK) were used to assess antimicrobial activity. To assess the antiproliferative activity of the polyphenolic extract, the following were used: fetal calf serum (FCS) and Dulbeco’s modified essential medium (DMEM, PAA Laboratories GmbH, Pashing, Austria), streptomycin (Galenika, Belgrade), trypsin (Serva, Heidelberg, Germany), EDTA (Laphoma, Skopje, North Macedonia), trichloroacetic acid, tris(hydroxymethyl)aminomethane, sulforodamine B (SRB), and dimethylsulfoxide (DMSO, 99.7%) (Sigma-Aldrich GmbH, Steinheim, Germany).

### 2.3. Ultrasound-Assisted Extraction of Polyphenols

To optimize the ultrasound-assisted extraction (UAE) of polyphenols from DRS, RSM was performed. The extractions were performed in the ultrasound bath EUP540A (Euinstruments, France). The examined parameters in the preliminary study were temperature (50–70 °C), time of extraction (15–45 min), solid/liquid ratio (10–30 mL/g), and ethanol concentration (60–100%). In total, 29 trials were performed using the Box–Behnken experimental design, and the optimal extraction conditions were: temperature 58 °C, time 15 min, liquid/solid ratio 17.8 mL/g, and 80% ethanol aqueous solution. The samples were stored at 4 °C.

### 2.4. Hydrolysis Procedure

To determine total EA content, hydrolysis was performed by the method of Määttä-Riihinen et al. [22] with some modifications: The DRS were homogenized and aliquots of 0.1 g were weighed and transferred into 5 mL laboratory flasks and diluted in 1 mL of 2 M HCl in methanol. Afterwards, acid hydrolysis was performed for 2 h at 85 °C with reflux. Then, the samples were quantitatively transferred into laboratory flasks, and 20 mL of 2 M HCl in methanol was added. The samples were sonicated for 30 min and filtered through a 0.45 µm membrane filter.

### 2.5. HPLC-DAD Analysis of Ellagic Acid

For the analysis of the EA content in the samples, the Agilent 1260 series HPLC-DAD system (Agilent Technologies, Santa Clara, CA, USA) was used with a C18 column (Agilent, 4.6 mm × 50 mm, 1.8 μm particles). The injection volume was 5 old, and the temperature was 30 °C. Solvent A was a 1% aqueous solution of formic acid, and solvent B was acetonitrile. The gradient used was as follows: 0–6 min, 15% of B; 6–28 min, 15–50% of B. The post run was set to 5 min. Good purity and separation were achieved in raspberry samples using this gradient (flow rate 0.5 mL/min). Ultraviolet-visible spectra (ranging from 190 to 540 nm) were recorded for all peaks. Triplicate analyses were performed for each sample. Ellagic acid was detected at 254 nm and identified according to peak retention time and UV/VIS spectra, which were compared with those of the standard. The quantities of ellagic acid were calculated using a calibration curve and expressed in mg/100 g [4]. The calibration curve coefficient of determination was R^2^ = 0.9983. The LOD was 0.0052 mg/mL of EA, and the LOQ was 0.0015 mg/mL of EA.

### 2.6. UHPLC-Triple-TOF-MS Analysis of Polyphenolic Profile

The polyphenolic profile of DRS extracts was determined using an ultra-high-performance liquid chromatography Symbiosis Pico UHPLC system. The detector used was a SCIEX TripleTOF 5600+ DuoSpray Source (TurboIonSpray and APCI). Data were analyzed using SCIEX MarkerView™, XCMSplus, MetaboAnalyst 4.0, and Analyst™ software. The samples (100 µL) were placed into 1.5 mL tubes, and then 800 µL of a 1:1 (*v*/*v*) mixture of acetonitrile and methanol was added. Vials were vortexed (2000 rpm for 15 min) and centrifuged at 13,000 rpm for 15 min. The supernatant was transferred to glass autosampler vials, which were then placed in an autosampler at 4 °C. The samples (8.0 µL) were injected directly into a Spark Holland Symbiosis™ Pico. Chromatographic separation was performed on a Hypersil chromatographic column, BDS C18, 150 × 4.6 mm, 5 mm, with a Hypersil C18 guard column (10 × 2.1 mm, size 5 μm). The mobile phase consisted of methanol:formic acid (99:1, *v*/*v*) A and water:formic acid (99:1, *v*/*v*) B, and the flow rate was constant: 500 µL/min. Gradient elution of mobile phase 100% A was started: 1.1–40 min linear gradient to 100% B, 40.1–55 min 100% B, and 55.1–60 min linear gradient to 100% A. The runtime of the method was 60 min. MS Parameters: The optimized detection conditions included curtain gas (N_2_) 25 psi, nebulizer gas (N_2_) 20 psi, heater gas (N_2_) 50 psi, ion source voltage floating 5500 V, and source temperature 500 °C. Samples were measured with a heated electrospray ionization probe in positive ionization mode (H-ESI+) with a declustering potential of 20 V and collision energy of 25 eV. The MS system was auto-calibrated using original calibrators (SCIEX) after every third analyzed sample. Every third analyzed sample was auto-calibrated using the Calibrant Delivery System (SCIEX).

### 2.7. Antioxidant Activity

#### 2.7.1. DPPH• Radical Scavenging Activity Assay

DPPH• antiradical activity was determined using an assay, as explained elsewhere [23]. Briefly, the methanol solution of the DPPH• radical was prepared and adjusted to reach an absorbance of 0.70 ± 0.02, using methanol. In total, 0.1 mL of the properly diluted extracts (1:60 *v*/*v*) was mixed with 2.9 mL of DPPH• radical solution. Afterwards, the samples were incubated at room temperature in a dark room for 60 min. Free radical scavenging measurements were performed at 517 nm (Shimadzu UV-1800, Kyoto, Japan). The results were reported as µmol of Trolox equivalents per g of sample (µmol TE/g).

#### 2.7.2. ABTS•^+^ Radical Scavenging Activity Assay

The method described by Usual et al. [24] was used to determine the antioxidant activity of extracts on ABTS•^+^ radicals. The ABTS reagent solution was prepared by mixing 7 mM aqueous 2,2′-azino-bis (3-ethylbenzothiazoline-6-sulfonic acid) diammonium salt and 2.45 mM potassium persulfate 1:1 (*v*/*v*) and incubating the mixture for 12 to 16 h at room temperature in the dark. After incubation, the reagent was diluted with acetate buffer (pH 3.3) to adjust the absorbance to 0.700 ± 0.02 at 734 nm (Shimadzu UV-1800, Kyoto, Japan). The extracts (0.1 mL) were mixed with the reagent (2.9 mL). The mixture was then set to incubate in a dark room at room temperature for 1 h, after which the absorbance at 734 nm was read. The results were expressed in μmol of Trolox equivalents per gram of sample (μmol TE/g of extract).

#### 2.7.3. Ferric Reducing Antioxidant Power (FRAP) Assay

Analysis of FRAP activity was conducted as described by Benzie and Strain [25]. A sample solution (1 mg/mL; 0.1 mL) was added to an FRAP reagent (2.9 mL) containing acetate buffer (0.3 M, pH 3.6), 2,4,6-tripyridyl-s-triazine (TPTZ) (10 mM) in 40 mM HCl, and iron(III) chloride (20 mM) in a ratio of 10:1:1 (*v*/*v*/*v*). After 1 h of incubation at room temperature, the absorbance of the sample at 593 nm was read. FRAP activity was expressed in μM Fe^2 +^ per g of extract (μM Fe^2+^/g extract).

### 2.8. Antibacterial Activity

The antibacterial activity of the raspberry seed extracts was tested against selected bacterial strains obtained from the American Type Culture Collection (Microbiologics, St Cloud, MN, USA): *Escherichia coli* (ATCC 25922), *Salmonella enterica* subsp. *enterica* serovar Enteritidis (ATCC 13076), *Staphylococcus aureus* (ATCC 25923), and *Listeria monocytogenes* (ATCC 19111). Reconstituted cultures were stored on agar slants in the refrigerator for one month and subcultured weekly onto a fresh nutrient agar (Himedia, Maharashtra, India) (total of 4 passages). After one month, they were safely discarded and a new commercial package (KWIK STIK) was reconstituted according to manufacturer (Microbiologics, St Cloud, MN, USA) instructions. The antibacterial assay was performed as described by Ledina et al. [26]. After overnight incubation on nutrient agar at 37 °C, well-isolated colonies of each test microorganism were inoculated into sterile saline and vortexed thoroughly. The density of the bacterial suspension was adjusted to the McFarland standard, 0.5 CFU/mL, using a DEN-1 densitometer (Biosan, Riga, Latvia). The antibacterial activity of the tested plant extracts was examined using the disk diffusion method. Mueller–Hinton agar (Himedia, Maharashtra, India) in sterile Petri dishes (90 mm) was inoculated with 100 μL of bacterial suspension containing 1–2 × 10^8^ CFU/mL using a sterile swab. The sterile filter paper disks (6 mm) impregnated with 10 μL of the plant extracts were placed on the agar surface. After 24 h of incubation at 37 °C, the plates were examined for inhibition zones. The control was an ethanol solution (80%, *v*/*v*) that showed a lack of antimicrobial activity due to its low added quantity and high microorganism concentration.

### 2.9. Antiproliferative Activity

#### Raspberry Seed Extracts and Ellagic Acid Standard

For the evaluation of antiproliferative activity, the extracts were dissolved and diluted in 9 mg/mL NaCl, sterilized using 0.22 μm syringe filters (Sartorius, Göttingen, Germany), and investigated in 0.39–200 μg/mL concentration range. EA (standard) was dissolved and diluted in DMSO and investigated in 0.244–62.50 μg/mL concentration range. Antiproliferative activity was evaluated in vitro in human cell lines: HeLa (cervix epithelioid carcinoma, ECACC No. 93021013), MCF7 (breast adenocarcinoma, ECACC No. 86012803), and MRC-5 (human fetal lung, ECACC No. 05090501), which was derived from healthy tissue. Cell lines were grown using previously described procedures [27]. The cell lines were harvested and plated into 96-well microtiter plates (Sarstedt, Newton, USA) at a seeding density of 4 × 10^3^ cells/well, in a volume of 180 μL (extracts) or 199 μL (standard), and pre-incubated in complete DMEM supplemented with 5% FCS, at 37 °C for 24 h. Serial twofold dilutions of the extracts (20 μL) or the standard (1 μL) were added to achieve the required final concentrations. Equal volumes of solvents (9 mg/mL NaCl or DMSO) were added to the control wells. The concentration of DMSO in the cell culture was ≤0.05% (*v*/*v*) [28]. Microplates were then incubated at 37 °C for 48 h. Cell growth was evaluated using the colorimetric SRB assay of Skehan et al. [29], modified by Četojevic-Simin et al. [30]. Color development was measured using a Multiscan Ascent (Labsystems; Helsinki, Finland) photometer at 540 nm against 620 nm as background. The effect on cell growth was calculated as 100 x (*A*_t_/*A*_c_) (%), where *A*_t_ is the absorbance of the test sample and *A*_c_ is that of the control. The concentration-cell growth (dose-effect) curves were drawn for each treatment, and IC_50_ values (concentration that inhibit cell growth by 50%) were determined using OriginPro 8 PRO software (Origin-Lab Corporation, Northampton, USA).

### 2.10. Statistical Analysis

All measurements were performed at least three times, and the results were expressed as mean ± standard deviation (SD). Analysis of variance (ANOVA) and post hoc Tukey’s HSD test at *p* < 0.05 significant level were used to examine significant statistical differences between polyphenolic extracts using STATISTICA 13.0 (StatSoft, Palo Alto, CA, USA).

## 3. Results and Discussion

### 3.1. Ellagic Acid Content

Free and total EA content in the DRS of all three examined cultivars are presented in Table 1. The results were obtained using UAE under optimal conditions for free EA, while total EA was determined for the samples obtained with hydrolysis.

Free EA content in the samples varied from 43.05 mg/100 g in DRS extracts of the Willamette variety to 46.76 mg/100 g in DRS extracts of the Meeker variety. The results suggest that there was a lack of statistically significant differences between free EA content in the Willamette and Polka varieties, while DRS from the Polka cultivar contained a slightly higher amount of free EA. A recent study reported that free EA content in Willamette was 45.23 mg/100 g dry weight (DW) when solid/liquid extraction (SLE) was combined with ethanolic solution (80% *w*/*w*) as solvent [31]. The free EA content in the mentioned study was comparable to the results obtained in the present study (46.76 mg/100 g) even though UAE was used as an extraction technique. This outcome could be explained by the longer extraction reported by Teslić et al. [31] compared to the present work (30 min vs. 15 min, respectively). This suggests the relevance of not only extraction technique selection but also extraction parameters. Similarly, there were differences in the EA content of raspberry seeds between the present study and the results (106.9 mg/100 g) reported by Majewski et al. [32]. Teslić et al. [31] also reported that the best-performing natural deep eutectic solvents (NADES) in terms of EA content were citric acid and betaine with a 3:1 molar ratio (147.02 mg/100 g DW) [31]. In the experiment with NADES, partial hydrolysis was performed with higher temperatures, extended extraction time, and favorable acidic conditions (citric acid) compared to the present study. However, the issue with this NADES extract could be the separation of EA from NADES. This separation would be required if EA needs to be added to food and cosmetic products, which will deteriorate with the addition of citric acid. Thus, ethanolic extract could be advantageous in this regard, since it is easier to evaporate aqueous ethanolic solution than to separate EA from NADES.

Total EA content was the highest for the Meeker cultivar (904.44 mg/100 g), while it was the lowest for cv. Polka (732.72 mg/100 g) (Table 1). This suggests that not only extraction techniques but also raspberry cultivars can influence EA content. Furthermore, which part of raspberry is submitted to extraction also plays an important role. Total EA content was significantly lower for Heritage, Autumn Bliss, Zeva, and Rubi cultivars when expressed in mg/kg of fruit fresh weight [33]. This is not a big surprise, since the major portion of EA is located in seeds [9], which represent only a small part of the raspberry fruit. Other studies have reported that total EA in Willamette ranges from 836.84 to 1031.41 mg/100 g [4,31], which is comparable to the present study.

### 3.2. Polyphenolic Profile

Over the past few years, researchers have focused on polyphenols contained in plant species [34,35]. These secondary plant metabolites, naturally present in fruits and vegetables, are part of the human diet. As effective free radical inhibitors, they potentially play a role in the prevention of human neurodegenerative diseases, cardiovascular disorders, and cancers. Polyphenols are compounds with a wide range of structures, from simple, containing one hydroxyl aromatic ring, to highly complex polymeric substances [36]. Therefore, knowledge of the polyphenolic profile of the extract is very important in terms of its use as a potential functional food supplement. Table 2, Table 3 and Table 4 present the phenolic profile of raspberry seed extracts from all three varieties. A total of 32 phenolic compounds were detected using UHPLC-Triple-TOF-MS in the seed extracts of the three varieties tested. Therefore, the significant biological activity of these extracts can be expected. Table 2 shows that the extracts contained various flavonoids, such as catechin, epicatechin, kaempferol, quercetin, and catechin dimer (procyanidin B). Besides, phenolic aglycones and some of their glycosides were detected (e.g., quercetin-hexoside and kaempferol-rhamnoside). The presence of flavonols in food affects food quality parameters, such as bitterness, acidity, sweetness, aroma, and color. The effect of flavonols on food functionality is reflected in the form of positive effects on health, i.e., flavonols have antioxidant, anticarcinogenic, antimicrobial, antiviral, and antifungal effects [37]. A recent study addressing polyphenols in raspberry seeds reported somewhat similar flavonoid profiles (e.g., catechin, epicatechin, procyanidin B1, quercetin, and kaempferol) [38]. Määttä-Riihinen et al. [22] analyzed soluble and insoluble polyphenolic compounds in red cultivated (cv. Muskoka), yellow cultivated, and red wild raspberry fruits and reported that there are differences in flavonoid profiles. For example, epicatechin was detected in all samples, while catechin was detected only in the yellow cultivated variety, suggesting the impact of raspberry variety on the flavonoid profile (Table 2).

Considering the flavonoids detected in UAE extracts, the same compounds were detected in extracts obtained from Polka and Meeker cultivars. On the other hand, some compounds, such as epicatechin, kaempferol, and quercetin-malonyl-hexoside were not detected in the Willamette extract recovered using UAE (Table 2).

Phenolic acids detected in DRS include caffeic, chlorogenic, gallic, ferulic, and EA (Table 3), whereas caffeic acid, ferulic acid, and EA were the only three phenolic acids detected in all samples. Phenolic acids are present in our diet in various foods, and due to their bioactive properties, there is evidence of their role in the prevention of autoimmune diseases. In addition, phenolic acids have been investigated for their antiallergic, antimicrobial, cardioprotective, anticancer, and antidiabetic properties [39,40]. All phenolic acids detected in our study have also been identified in Harigate cv. raspberry seeds [38]. On the other hand, ferulic acid and caffeic acid have not been detected in the red cultivated (cv. Muskoka), yellow cultivated, and red wild raspberry fruits [22].

The results suggest that there were no differences between the phenolic acid profiles of the Willamette and Polka cultivars extracts obtained using UAE under optimal conditions, while chlorogenic acid was detected only in the Meeker UAE sample (Table 3). Regarding hydrolysis samples, coumaric acid was observed only for cv. Willamette, while gallic acid was not detected in cv. Meeker.

The importance of anthocyanins in the human diet is reflected in the fact that people who consume larger amounts of foods rich in anthocyanins have a reduced risk of developing cardiovascular disease [41]. Anthocyanins and anthocyanidins were also present in all extracts obtained, which was rather expected since particularly hydrolysis extracts were red. The presence of anthocyanins and anthocyanidins in extracts that are responsible for the color of various fruits, including raspberries, could originate from fruit pulp residuals that are attached to seeds. Anthocyanidins and their glycosylated forms identified in DSR are shown in Table 4. Aglycone pelargonidin and cyanidin-3-O-glucoside were identified in all samples, while cyanidin-hexosil-pentoside was not present in the cv. Meeker extract obtained using UAE and cyanidin-rhamnetin-dihexoside was not present in the cv. Polka hydrolysis extract. Veberic et al. [42] reported the presence of pelargonidin in cultivated and wild grown raspberry, while Määttä-Riihinen et al. [22] and Veberic et al. [42] identified several pelargonidin and cyanidin glycosides in red raspberry fruits.

### 3.3. Antioxidant Activity

To assess overall antioxidant activity, several in vitro antioxidant tests have been developed. Although these methods are limited in terms of their similarity to the mechanisms of antioxidant effects in the biological system, they may well demonstrate how polyphenols function as antioxidants. Anyhow, caution must also be exercised when assessing the antioxidant activity of in vitro models because they do not take into account the metabolic transformations and interactions known to affect the biological properties of polyphenolic compounds. From the point of view of chemistry, polyphenol molecules, after donating an electron or hydrogen atom, themselves become free radicals, and if they are present in sufficient quantities, they can potentially cause pro-oxidant activity. However, the question is whether such pro-oxidant activity will occur in in vivo systems and be beneficial for human health via pro-apoptotic effect towards some types of cancer cells or will it cause harm to humans via formation of reactive oxygen species (ROS), which are potentially cytotoxic for healthy tissues as well. In any case, this indicates the need for further research [43].

In vitro antioxidant activity (DPPH, ABTS, and FRAP assays) of the samples obtained by UAE is shown in Table 5.

Regarding the DPPH test, the highest antiradical scavenging effect was observed for the Willamette extract (359.76 µmol TE/g), while the antioxidant activity of the extracts from the other two varieties was significantly lower. The extract obtained from cv. Willamette compared to the other two varieties showed a significantly lower antioxidant effect when analyzed using ABST and FRAP assays. Taking into account previous research examining the role of polyphenolic compounds as antioxidants that protect against the most common diseases associated with oxidative stress, such as cardiovascular disease, inflammation, cancer, and neurodegenerative diseases, polyphenols from fresh berries and berry products (e.g., dietary supplements) may play a useful role as antioxidants in humans [44]. On the other hand, several studies have reported that phenolic compounds could also have pro-oxidant activity, which could potentially have a negative impact on human health [43], as mentioned earlier. Certain antioxidant activity of raspberry seed extract was expected since a relatively large number of polyphenols was detected using UHPLC-Triple-TOF-MS in these extracts (Table 2, Table 3 and Table 4). Some of the detected polyphenols, such as EA, quercetin, catechin, and epicatechin, are well-known antioxidants [5,45,46]. In a recent study, it was reported that catechins (which were also present in raspberry seeds extracts) have high antioxidant potential compared to other flavonoids (e.g., naringin, rutin, hesperidin), hydroxycinnamic acids (e.g., chlorogenic acid, caffeic acid, hydrocinnamic acid.), and other synthetic (e.g., ascorbic acid) and natural antioxidants (e.g., curcumin) [46]. The only quantified polyphenol in the present study was free EA (43.05–46.76 mg/100 g) and total EA (732.72–902.44 mg/100 g). Even though there is a significant statistical difference between cv. Meeker compared to the other two varieties, the differences in absolute value were minimal. This suggests that free EA content has a low impact on the differences in, e.g., ABTS cation scavenging activity, which is approximately twofold higher for cv. Meeker compared to cv. Willamette (Table 5). On the other hand, total EA, which is generally presented as bonded in the form of ellagitannins, is significantly higher in cv. Meeker (902.44 mg/100 g) compared to cv. Polka (732.72 mg/100 g), while their antioxidant activity determined using the ABTS test is partially equal (Table 5). All this indicates that other polyphenols in raspberry seed extracts contributed greatly to antioxidant activity in the samples. Considering the research conducted by Park et al. [47], who examined black raspberry seed extracts using the FRAP assay, the high antioxidant activity of this matrix has been proven (1041.0 ± 97.8 µM TEAC/g). Due to different units and protocols, it is not always possible to straightforwardly compare the results of antioxidant activity tests between studies [48]. Koca and Karadeniz [49] reported that the extracts obtained from blackberries and blueberries fruits have a similar or lower ferric reducing antioxidant power effect (7.41–0.41 µmol/g) than the raspberry seed extracts in the present study (71.98–118.30 µmol Fe^2+^/g). Since the former is most likely expressed as fruit fresh weight, while the latter is expressed as seed dry weight, such an outcome could be possible. Teslić et al. [31] reported that the DPPH activity of obtained cv. Willamette extract is 197.78 ± 2.76 μmol TE/g DW when 80% ethanol and SLE are used to extract phenolic compounds from raspberry seeds. This suggests that a larger quantity of polyphenols with higher antioxidant activity was extracted using UAE compared to trials from a previous study [31]. Furthermore, the DPPH radical scavenging potential of the extract obtained using hydrolysis is significantly higher (4128.92 ± 35.91 μmol TE/g DW) compared to the results obtained in the present study (Table 5). This indicates that products obtained using hydrolysis exhibit high antioxidant activity.

### 3.4. Antibacterial Activity

Extracts obtained from the DRS of all three varieties were subjected to determination of potential antibacterial activity. Antibacterial activity against two Gram-negative (*E. coli* and *S. enteritidis*) and two Gram-positive bacterial strains (*S. aureus* and *L. monocytogenes*) was determined, and the results are shown in Table 6.

Polyphenolic extracts from all three raspberry cultivars showed some antimicrobial activity against all tested bacterial strains. However, the results obtained imply a low antimicrobial activity, since all zones of inhibition were less than 15 mm [50]. Thus, raspberry seed extract could not be used as an effective antimicrobial agent. Comparing the examined cultivars, the Willamette extract had significantly higher antimicrobial activity compared to the extracts obtained from Meeker and Polka seeds, but was still insufficiently effective to be considered as an antimicrobial agent. Some antimicrobial activity was expected from raspberry seed extract since EA was detected in all samples (Table 1). According to Ghudhaib et al. [51], EA showed a certain inhibition zone towards *Staphylococcus epidermidis* (7–20 mm), *Bacillus cereus* (8–19 mm), *Klebsiella pneumoniae* (9–19 mm), and *Salmonella typhi* (8–17 mm), which was concentration dependent. On the other hand, pomegranate extract with a significant amount of EA (345 mg/g extract) did not inhibit *E. coli* growth in strawberry juice when pomegranate extract was added in a concentration of 360 μg/mL juice [52].

Cvetnić and Vladimir-Knezevic [53] suggested that grapefruit seed extracts, which also contain flavonoids, manifest some antimicrobial effects towards various microorganisms. The inhibition zone caused by ethanolic grape seed extracts ranged from 10 to 16 mm and 9 to 13 mm towards 10 Gram-positive bacteria strains (including *Staphylococcus aureus* and *Listeria monocytogenes*) and 10 yeast strains, respectively, while 10 Gram-negative bacteria strains, including *Salmonella enteritidis* and *Escherichia coli*, were resistant to these extracts [53].

### 3.5. Antiproliferative Activity

Antiproliferative activity of isolated UAE and hydrolysis extracts of all three cultivars was evaluated using cervical carcinoma (HeLa), breast adenocarcinoma (MCF7), and fetal lung (normal cells; MRC-5) human cell lines. The results are shown in Table 7. The influence of extracts on cell growth depended on the extraction method, variety, and cell line. For both types of extraction, the lowest IC_50_ values, i.e., the most pronounced antiproliferative effect, were obtained for the MCF7 cell line. The best-performing extract obtained using UAE was from the Meeker variety, with the lowest IC_50_ values for all investigated cell lines (IC_50_^MCF7^ = 4.92 μg/mL, IC_50_^HeLa^ = 5.19 μg/mL and IC_50_^MRC-5^ = 8.24 μg/mL). The most active extract obtained after hydrolysis was from cv. Willamette, with the lowest IC_50_ values for all examined cell lines (IC_50_^MCF7^ = 9.32 μg/mL, IC_50_^HeLa^ = 31.22 μg/mL and IC_50_^MRC-5^ = 22.33 μg/mL) (Table 7). Thus, raspberry seed ethanol extracts, particularly from the Meeker variety, are good candidates for use as functional ingredients with high antiproliferative activity. In particular, the extracts are more lethal towards MCF7 cancer cells compared to MRC-5, which are considered normal cells (Table 7). This indicates that raspberry seed extracts could have some therapeutic window in which tumor tissues (MCF7) would have a higher death rate compared to healthy tissues (MRC-5).

Since the yield of EA was significantly higher after hydrolysis compared to UAE (Table 1), it was rather expected that hydrolysis extracts would perform better in terms of antiproliferative activity, particularly since the EA standard showed exceptional antiproliferative activity for all evaluated cell lines (IC_50_^MCF7^ = 11.02 μg/mL, IC_50_^HeLa^ = 2.47 μg/mL and IC_50_^MRC-5^ = 3.43 μg/mL). Surprisingly, raspberry seed extracts obtained using UAE had significantly higher antiproliferative activity compared to extracts obtained using hydrolysis for all evaluated cell lines (Table 7). The reason for this may be the degradation of polyphenols with antiproliferative activity (not detected with UHPLC-Triple-TOF-MS) during hydrolysis due to their instability at higher temperatures and in an acidic medium. Furthermore, the EA standard had a higher IC_50_ value (11.02 µg/mL) for the MCF7 cell line compared to the Meeker UAE sample (IC_50_ = 4.92 µg/mL), which suggests higher antiproliferative activity of raspberry seed extract (Table 7). This may be due to the presence of other compounds with antiproliferative activity in raspberry seed extract [54], which can act synergistically, resulting in higher antiproliferative activity of extracts compared to individual components. Compounds that could contribute to the antiproliferative activity of extracts are, e.g., gallic acid and quercetin [11], both detected in the majority of the obtained samples (Table 2, Table 3 and Table 4). The results of this study suggest that the type of extraction procedure, along with the difference in polyphenolic content and phenolic profile, also has a significant influence on the level of antiproliferative activity of the obtained extracts.

Other studies have also reported the antiproliferative activity of 80% ethanolic raspberry seed extract (cv. Willamette) toward human Caucasian colon adenocarcinoma cells (IC_50_ 22.93–61.82 mg/mL) [31]. Antiproliferative activity of cv. Willamette and cv. Meeker pomace extracts (pulp with seeds) obtained using 80% methanol with 0.05% acetic acid as solvent [11] was lower (IC_50_^MCF7^ = 34.9–60.3 μg/mL, IC_50_^HeLa^ = 57.3–73.1 μg/mL and IC_50_^MRC-5^ = 129–149 μg/mL) compared to seed extract activity (Table 7). This indicates that DRS are a more abundant source of bioactives from raspberry food production by-products. In the same study, the authors reported that standard solutions of commercial chemotherapeutics with strong antiproliferative effects, such as Doxorubicin^®^ (0.25–0.40 μg/mL) and Gemcitabine^®^ (0.04–0.13 μg/mL), are significantly higher compared to raspberry pomace extracts [11].

The antiproliferative activity of polyphenols could be expressed through several mechanisms. One of the mechanisms is correlated with inflammatory processes. These processes can be prevented by reducing oxidative stress conditions via antioxidant agents. Polyphenols and their aromatic rings react with ROS and reactive nitrogen species (RNS), which are formed as a consequence of abnormal metabolic reactions. The ROS and RNS thus formed could lead to the transformation of inflammatory tissues into cancer cells [55]. Thus, if polyphenols are consumed in sufficient quantity, they could potentially reduce the risk of carcinogenesis via their antioxidant activity. Another mechanism could be related to the ability of plant polyphenols to mobilize endogenous copper ions and thus prevent/reduce oxidative DNA breakage in human cells [56], which could also lead to carcinogenesis. Besides cancer prevention, polyphenols could also induce apoptosis (programmed cell death) in cancer cells through several mechanisms [57]. One of these mechanisms is the initiation of H_2_O_2_ production in tumor cells, which increases oxidative stress and damages DNA in tumor cells [57]. Therefore, polyphenols and plant extract could have a noticeable role in cancer prevention and cancer treatment.

## 4. Conclusions

The experimental results lead to the conclusion that the DRS from the three cultivars examined contain a wide spectrum of polyphenols, such as phenolic acids and flavonoids. EA content was significantly higher in hydrolysis extracts compared to ethanolic extracts, indicating that the majority of EA is in bonded form. The results suggested that extracts have high in vitro antioxidant properties, high antiproliferative bioactivity, and low antimicrobial activity. The extracts from the Meeker variety exhibited the highest antioxidant activity towards DPPH• radical scavenging (336.38 µmol TE/g) and ferric reducing antioxidant power (118.30 µmol Fe^2+^ µmol/g), while extracts from the Polka variety had the strongest activity when analyzed with the ABTS assay (434.87 µmol TE/g). Moreover, the UAE extracts of the Meeker variety exhibited the highest antiproliferative activity in comparison to the other two cultivars (4.92–8.24 µg/cm^3^). It can be concluded that examined seeds, which are by-products of raspberry food production, can be utilized as a highly valuable and inexpensive raw material for the production of pharmaceuticals, functional food products, and dietary supplements.

## Figures and Tables

**Table 1:** Free and total EA content in the DRS of all examined varieties.

Variety	Free EA (mg/100 g) *	Total EA (mg/100 g) **
Willamette	43.05 ± 0.59 ^b^	859.11 ± 5.45 ^b^
Polka	44.29 ± 0.17 ^b^	732.72 ± 7.75 ^c^
Meeker	46.76 ± 1.03 ^a^	902.44 ± 7.12 ^a^

The results are presented as mean ± SD, *n* = 3. Different letters (a, b, and c) within the same column indicate significant statistical differences according to Tukey’s HSD test (*p* < 0.05). * Obtained with UAE under optimal conditions. ** Obtained with hydrolysis.

**Table 2:** Flavonoids detected in extracts of defatted Willamette, Polka, and Meeker raspberry seeds, obtained using UAE under optimal conditions and hydrolysis.

Compound	Retention Time (min)	Precursorion (*m*/*z*)	Production (*m*/*z*)	Sample					
				1	2	3	4	5	6
Catechin	25.30	139.0792	123.1212	d	d	d	d	d	d
Catechin dimer (Procyanidin B)	26.32	289.0438	125.6281	d	d	d	d	d	d
Kaempferol	16.32	487.2653	287.1222	d	d	d	nd	d	d
Kaempferol-glucuroside-diramnoside	15.56	588.8521	215.5251	d	d	d	d	d	d
Kaempferol-glucuronide	14.32	593.4080	285.3694	d	d	nd	d	d	d
Kaempferol-malonyl hexoside	16.56	515.0803	274.0121	d	d	d	d	d	d
Kaempferol-rhamnosil dihexoside	17.01	515.1379	325.3231	d	d	nd	d	d	d
Kaempferol-dihexoside	16.44	518.1514	287.2852	d	d	nd	d	d	d
Kaempferol-rhamnoside	15.32	575.2322	252.0191	d	d	d	d	d	d
Epicatechin	10.32	289.5685	245.0842	d	d	d	nd	d	d
Fisetin	18.65	213.4379	139.0087	d	d	d	d	d	d
Myricetin	19.65	151.2469	108.3229	d	d	d	d	d	d
Naringenin	21.32	235.4685	124.5924	d	d	d	d	d	d
Quercetin	36.39	301.6185	151.8237	d	d	d	d	d	d
Quercetin-arabinoside	36.85	355.3487	147.3417	d	d	d	d	d	d
Quercetin-dihexoside	35.63	345.1283	175.1474	d	d	d	d	d	d
Quercetin-3-(6-o-galloylgalactoside)	34.52	365.1364	185.3449	d	d	d	d	d	d
Quercetin-glucuronide	33.32	385.0433	201.0062	d	d	d	d	d	d
Quercetin-hexoside	36.35	355.2776	198.1954	d	d	d	d	d	d
Quercetin-malonyl-hexoside	34.52	365.0222	187.7822	nd	d	d	nd	d	d
Rhamnetil-glucuronide	45.12	420.1541	179.6961	d	d	d	d	d	d
Rhamnetil-malonyl-hexoside	44.36	485.0097	198.0891	d	d	d	d	d	d

nd—not detected; d—detected; 1—hydrolysis extract of the Willamette variety seeds; 2—hydrolysis extract of the Polka variety seeds; 3—hydrolysis extract of the Meeker variety seeds; 4—UAE extract of the Willamette variety seeds; 5—UAE extract of the Polka variety seeds; 6—UAE extract of the Meeker variety seeds.

**Table 3:** Phenolic acids detected in extracts of defatted Willamette, Polka, and Meeker raspberry seeds, obtained using UAE under optimal conditions and after hydrolysis.

Compound	Retention Time (min)	Precursor Ion (*m*/*z*)	Product Ion (*m*/*z*)	Sample					
				1	2	3	4	5	6
Chlorogenic acid	15.65	355.2802	163.9814	d	d	d	nd	nd	d
Caffeic acid	36.32	181.8385	163.9882	d	d	d	d	d	d
Coumaric acid	16.32	103.0878	123.3232	d	nd	nd	nd	nd	nd
Ellagic acid	18.63	165.1013	101.3014	d	d	d	d	d	d
Ferulic acid	9.56	149.5521	139.1481	d	d	d	d	d	d
Gallic acid	13.32	127.5114	109.1745	d	d	nd	d	d	d

nd—not detected; d—detected; 1—hydrolysis extract of the Willamette variety seeds; 2—hydrolysis extract of the Polka variety seeds; 3—hydrolysis extract of the Meeker variety seeds; 4—UAE extract of the Willamette variety seeds; 5—UAE extract of the Polka variety seeds; 6—UAE extract of the Meeker variety seeds.

**Table 4:** Anthocyanins and anthocyanidins detected in extracts of defatted Willamette, Polka, and Meeker raspberry seeds, obtained using UAE under optimal conditions and after hydrolysis.

Compound	Retention Time (min)	Precursorion (*m*/*z*)	Production (*m*/*z*)	Sample					
				1	2	3	4	5	6
Cyanidin-3-O-glucoside	50.25	449.1253	128.6344	d	d	d	d	d	d
Cyanidin-hexosil-pentoside	32.52	402.0451	125.5948	d	d	d	d	d	nd
Cyanidin-rhamnetin-dihexoside	36.32	356.1842	109.1251	d	nd	d	d	d	d
Pelargonidin	23.25	356.0011	241.2268	d	d	d	d	d	d

nd—not detected; d—detected; 1—hydrolysis extract of the Willamette variety seeds; 2—hydrolysis extract of the Polka variety seeds; 3—hydrolysis extract of the Meeker variety seeds; 4—UAE extract of the Willamette variety seeds; 5—UAE extract of the Polka variety seeds; 6—UAE extract of the Meeker variety seeds.

**Table 5:** Antioxidant activity of the polyphenolic extracts of all the examined raspberry varieties, obtained using UAE under optimal conditions.

Cultivar	DPPH (µmol TE/g)	ABTS (µmol TE/g)	FRAP (µmol Fe^2+^/g)
Willamette	359.76 ± 17.56 ^a^	217.38 ± 3.01 ^b^	71.98 ± 3.0 ^b^
Polka	330.76 ± 25.99 ^b^	434.87 ± 18.74 ^a^	117.12 ± 1.89 ^a^
Meeker	336.38 ± 3.69 ^b^	430.29 ± 4.64 ^a^	118.30 ± 2.03 ^a^

The results are presented as mean ± SD, *n* = 3. Different letters (a and b) within the same column indicate significant statistical differences according to Tukey’s HSD test (*p* < 0.05).

**Table 6:** Antimicrobial activity of the polyphenolic extracts of all the examined raspberry varieties, obtained using UAE under optimal conditions.

Cultivar	Growth Inhibition Zone of Four Bacterial Strains (mm)			
	*S. aureus*	*E. coli*	*L. monocytogenes*	*S. enteritidis*
Willamette	9.00 ± 1.63 ^a^	10.00 ± 0.00 ^a^	9.33 ± 0.47 ^a^	9.00 ± 0.00 ^a^
Meeker	7.33 ± 0.47 ^a^	7.00 ± 0.00 ^b^	7.33 ± 0.47 ^b^	7.33 ± 0.47 ^a^
Polka	8.67 ± 0.47 ^a^	7.00 ± 0.00 ^b^	7.33 ± 1.25 ^b^	8.50 ± 0.50 ^a^

The results are presented as mean ± SD, *n* = 4. Different letters (a and b) within the same column indicate significant statistical differences according to Tukey’s HSD test (*p* < 0.05).

**Table 7:** Antiproliferative activity of the polyphenolic extracts of all examined raspberry varieties, obtained using UAE under optimal conditions and after hydrolysis.

Extraction Method	Variety	IC_50_ (µg/mL)		
		HeLa	MCF7	MRC-5
UAE	Willamette	12.12 ± 2.67 ^c2^	6.47 ± 0.07 ^bc1,2^	11.11 ± 1.42 ^c1^
	Polka	10.29 ± 2.87 ^c2^	8.19 ± 2.42 ^b2^	8.98 ± 1.20 ^c1^
	Meeker	5.19 ± 0.49 ^d1^	4.92 ± 0.70 ^c1^	8.24 ± 1.41 ^c1^
Hydrolysis	Willamette	31.22 ± 5.58 ^b1^	9.32 ± 1.65 ^b1^	22.33 ± 2.26 ^b1^
	Polka	67.69 ± 7.79 ^a2^	30.66 ± 7.59 ^a2^	35.93 ± 10.24 ^a2^
	Meeker	69.43 ± 7.48 ^a2^	32.49 ± 7.97 ^a2^	26.72 ± 3.63 ^b1^
Standard	EA	2.47 ± 0.40 ^d^	11.02 ± 0.75 ^b^	3.43 ± 0.08 ^d^

The results were presented as mean ± SD, *n* = 4. Different numbers (1 and 2) in the same column indicate significant statistical differences between cultivars (extraction technique = const.), and different letters (a, b, c, and d) in the same column indicate significant statistical differences between the applied extraction techniques according to Tukey’s HSD test (*p* < 0.05).

## Data Availability

The data used to support the findings of this study can be made available by the corresponding author upon request.
